# Global burden of age-related macular degeneration (1990–2021): trends, age-sex disparities, and socioeconomic dynamics from the GBD study

**DOI:** 10.3389/fpubh.2025.1594672

**Published:** 2025-10-23

**Authors:** Haorong Kong, Hui Feng, Hong Wang

**Affiliations:** ^1^Beijing Tongren Hospital, Capital Medical University, Beijing, China; ^2^Beijing Key Laboratory of Ophthalmology and Visual Sciences, Beijing, China; ^3^Beijing Tongren Eye Center, Beijing, China

**Keywords:** age-related macular degeneration, trends, disparities, global burden of disease study, prevalence, years lived with disability

## Abstract

**Objectives:**

This study aimed to assess the global burden of Age-related macular degeneration (AMD) across countries, regions, and age groups by sex, sociodemographic index (SDI) level, and risk factors from 1990 to 2021, using newly updated data from the Global Burden of Disease (GBD) study. The focus was on age-related disparities in AMD burden by sex and SDI.

**Methods:**

This population-based study utilized AMD data from GBD 2021 (1990–2021). The burden was evaluated using the number of cases, prevalence rates per 100,000 population, and trends in years lived with disability (YLDs) and prevalence, assessed through average annual percentage changes (AAPCs) and estimated annual percentage changes (EAPCs).

**Results:**

Globally, AMD prevalence increased from 364,000 cases in 1990 to 806,000 in 2021 (+121%), while YLDs rose from 30,000 to 58,000 (+91%). However, age standardized prevalence and YLD rates (ASPRs and ASYRs) significantly declined (EAPCs: −0.26 and −0.94, respectively). Regional analyses revealed that low SDI regions (e.g., sub-Saharan Africa) bore the highest AMD burden (ASPR: 139.9 per 100,000) and exhibited a younger age distribution, with a significantly higher proportion of cases in individuals aged 55–74 years. In contrast, high SDI regions (e.g., high-income Asia-Pacific) had a concentration of cases among those aged 70 years or older. While medium SDI regions accounted for one-third of global cases in 2021, age-standardized rates declined most slowly in low SDI regions (EAPC: −1.03) and even trended upward in some countries. AMD cases peaked globally at ages 65–69, yet prevalence was highest among those over 85 years (1,349.9 per 100,000), with women generally experiencing a higher burden than men. These findings highlight distinct regional patterns, with younger disease profiles in low SDI regions and aging-driven increases in high SDI regions, underscoring the need for targeted prevention and control strategies.

**Conclusion:**

Although global efforts over the past 30 years have led to a decline in AMD prevalence rates and YLDs, the absolute number of cases and YLDs continues to rise, driven by age, sex, socioeconomic status, and geographic location. These findings provide an epidemiological basis for developing global public health strategies to address these ongoing challenges.

## 1 Introduction

Age-related macular degeneration (AMD) is a debilitating retinal disease that progressively impairs vision, primarily targeting the macular. Ranking third globally in blindness among the older adults after cataracts and glaucoma, it is a pressing concern as the leading cause of severe vision loss among individuals over 55 years old (1). This disease damages the macula and threatens central vision, which is vital for essential activities like reading, driving, and recognizing face. Consequently, AMD severely diminishes quality of life, undermines independence, and increases risks of falls, depression, and social isolation (2). In 2019, the global prevalence of AMD reached an alarming 96.76 per 10,000 people highlighting the immense health and economic toll of this condition (3). High disease burdens are usually caused by socio-demographic underdevelopment, limited accessibility and poor healthcare systems (4). For example, in developing countries such as Bhutan, 45% of AMD patients present with late-stage disease, and half of wet AMD cases already have disciform scars at first presentation, indicating delayed diagnosis and limited treatment availability (5). Besides, wet AMD imposes huge economic burden on patients and healthcare systems as the common indication for anti-VEGF injection (6). However, in many high middle SDI regions, AMD still causes a significant burden of disease (7). Besides with an aging global population, the number of AMD cases continues to rise, signaling a growing public health crisis that demands immediate action (8). Addressing AMD is not merely a medical necessity but a societal imperative to safeguard the well-being and autonomy of an increasingly aging population. While previous studies have explored the burden of AMD, many relied on outdated data, focused on specific countries or regions, or examined the impact of isolated risk factors (9, 10), few studies have described epidemiologic trends in AMD analyzing age-sex multidimensionality, such as the burden of disease for AMD by age across sex or SDI levels.

The Global Burden of Disease (GBD) study offers policymakers invaluable insights into long-term health trends at global, regional, and national levels by consistently updating its epidemiologic databases. GBD 2021 provides the most recent and comprehensive data on AMD, covering prevalence, years lived with disability (YLDs) and associated risk factors in 204 countries and territories and 811 subnational locations (11). Given the chronic, progressive nature of AMD, which is characterized by irreversible structural changes, early and aggressive management is crucial to slow disease progression. To advance global understanding of AMD epidemiology, this study utilized data from GBD 2021 to deliver an up-to-date, comprehensive assessment of AMD’s burden, trends, and inequities. Specifically, the analysis includes: (1) a descriptive evaluation of AMD epidemiology at global, regional, and national levels; (2) multidimensional convergence analysis based on age, gender, and socio-demographic index (SDI); and (3) Joinpoint regression analysis to identify temporal trends in the data.

## 2 Methods

### 2.1 Data source and disease definition

The AMD data analyzed in this study are sourced from the GBD 2021, All this data is accessible for free access through the Global Health Data Exchange.^1^ The GBD 2021 project estimated the rates, numbers and percentages change of incidences, prevalences, deaths and disability adjusted life years (DALYs) for 371 diseases and injuries in 204 countries and territories and 811 subnational locations (11). Detailed descriptions of these indices are included in the appendix of the GBD 2021 capstone paper. To ensure consistent and reliable estimates of disease burden, the project employs the Disease Model Bayesian Meta-Regression (DisMod-MR) tool (version 2.1). This tool leverages the Bayesian Priors, Regularization, and Trimming (MR-BRT) framework to integrate all available morbidity and mortality data, epidemiological relationships, and spatial correlations. The general methods and disease burden estimation methods of GBD 2021 have been previously reported (11). By applying this rigorous methodology, GBD 2021 delivers highly consistent and accurate estimates of AMD’s global burden, supporting informed decision-making and evidence-based policy development.

### 2.2 Descriptive analysis

To achieve a comprehensive understanding of AMD’s burden, a descriptive analysis was conducted at the global, regional, and national levels. This analysis visually represented the global case numbers, crude rates, and age-standardized rates (ASRs) of prevalence (ASPR) and Years Lived with Disability (YLDs; ASYR) for AMD, disaggregated by sex (both sexes, males, and females) from 1990 to 2021. The methodology for calculating YLDs, which involves multiplying disease prevalence by its corresponding disability weight, has been described in previous studies. The increase in YLDs reflects the aging global population, corresponding to the decline in mortality. Thus, instead of DALYs, YLDs may be more valuable for prevalence assessment and policymaking in the evaluation of AMD burden (12, 13). Additionally, a comparative analysis was performed to examine changes in case numbers and ASRs of prevalence and YLDs for AMD between 1990 and 2021. This analysis covered global, regional (21 GBD-defined geographical regions), and national (204 countries and territories) levels, as well as comparisons across five socio-demographic index (SDI) quintiles: high, high-middle, middle, low-middle, and low. SDI is a composite metric that captures socio-demographic development by incorporating three factors: the total fertility rate of the population under 25, the average educational attainment of individuals aged 15 and older, and the lagged income distribution index per capita. These analyses provide critical insights into the evolving epidemiological landscape and inequities in AMD burden across different contexts.

### 2.3 Trend analysis

Investigating changes in disease trends is a critical aspect of epidemiology, enabling the development of targeted prevention strategies. This study aimed to analyze trends in AMD from overall, local, and multidimensional perspectives. To assess the overall trend, we used percentage change (PC) and estimated annual percentage change (EAPC). PC quantifies the percentage change in the absolute number of AMD cases or YLDs between two points, while EAPC measures the annual trend in rates. It requires standardization to account for differences in age structures or changing age profiles within groups when comparing several groups with distinct age structures or one certain group with age profile changing over time. Among these metrics, EAPC provides a more robust indicator for monitoring shifts in disease patterns over time (14). The ASR was classified as increasing if both the EAPC estimate and its 95% uncertainty Interval (UI) lower bound were greater than 0, decreasing if both the EAPC estimate and its 95% UI upper bound were less than 0, or stable otherwise. The formulas for calculating EAPC and PC are provided.

To identify localized trends in AMD burden, we employed Joinpoint regression analysis. Joinpoint regression analysis was performed using the Joinpoint software (version 5.4.0) to assess temporal trends ASPR and ASYR for AMD from 1990 to 2021. To ensure models achieved maximum complexity while maintaining sufficient degrees of freedom for each segment, we capped the maximum number of joinpoints at 4. Starting with the simplest model (0) joinpoints, the software incrementally increased the number of joinpoints (0, 1, 2, 3, 4) using permutation tests and compared model fit. To control Type I error inflation from multiple comparisons, the significance levels of permutation tests underwent Bonferroni correction. The final model selected was the most complex statistically significant model. For this chosen model, we documented the joinpoint locations (years) of connection points and their 95% confidence intervals, the APC and its 95% CI for each segment, and the overall AAPC across the study period. Since the GBD database provides 95% uncertainty intervals (UI) rather than direct standard errors (SE), we estimated SE based on the UI and inputted it as “Standard Error into Joinpoint. Subsequently, Joinpoint generated 95% confidence intervals for APC and AAPC using weighted least square (15). If the lower bounds of both the APC/AAPC estimates and their 95% CI were greater than 0, an upward trend was identified. Conversely, if the upper bounds of both estimates were less than 0, a downward trend was determined. Trends were deemed stable if neither condition was met. This comprehensive approach provides valuable insights into the dynamic patterns of AMD burden across time and regions.

## 3 Results

### 3.1 Global level

**Figure 1:**
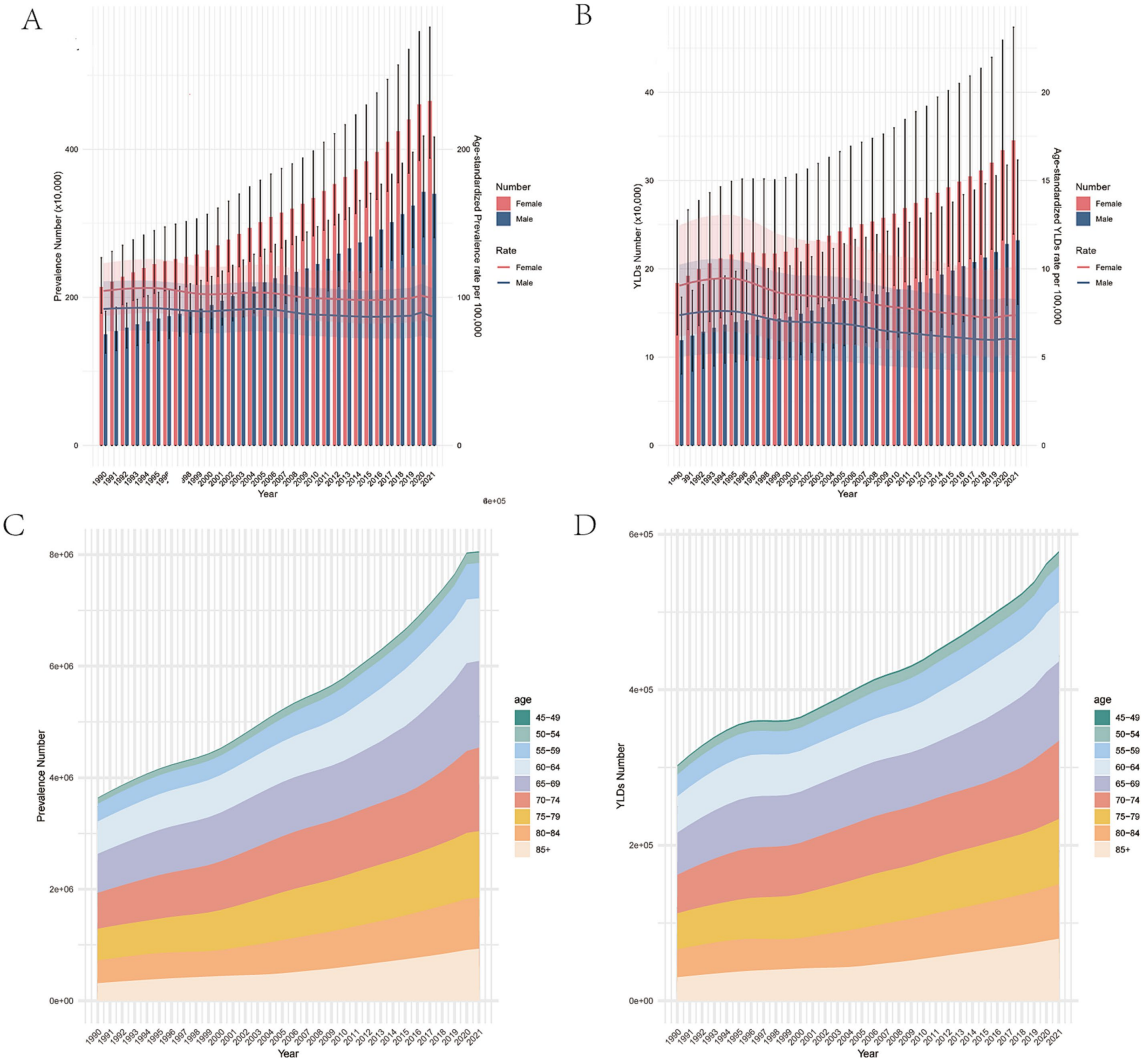
Changes in global burden of the prevalence and YLDs of AMD from 1990 to 2021. **(A)** The number of cases and age-standardized prevalence rates of AMD from 1990 to 2021. **(B)** The number of YLDs and age-standardized YLD rates of AMD from 1990 to 2021. **(C)** The number of prevalent cases of AMD in different age subgroups from 1990 to 2021. **(D)** The number of YLDs cases of AMD in different age subgroups from 1990 to 2021. Red and blue dashed line indicates the upper and lower limits of the 95% uncertainty intervals (95% UIs) for females and males, respectively. AMD, age-related macular degeneration; UI, uncertainty interval; YLDs, years lived with disability.

**Figure 2:**
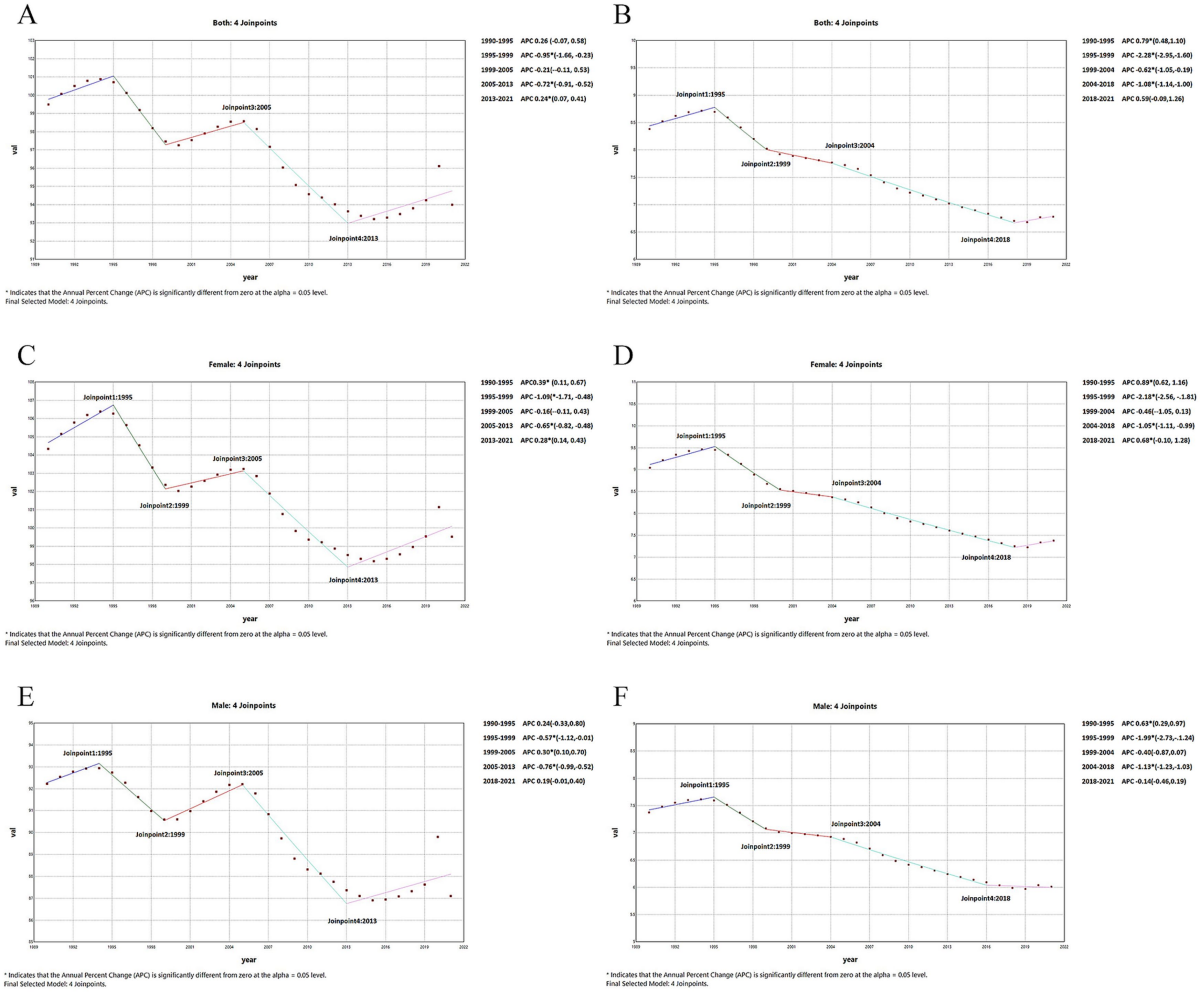
Global changes of ASR from 1990 to 2021by sex. **(A)** ASPR of females. **(B)** ASYR of females. **(C)** ASPR of males. **(D)** ASYR of males. **(E)** ASPR of both sexes. **(F)**. ASYR of both sex. ASR, age-standardized rates; ASPR, age-standardized prevalence rates; ASYR, age-standardized YLDs rates; AMD, age-related macular degeneration.

The global trends in prevalence and YLDs for AMD from 1990 to 2021 are presented in Table 1 and Supplementary Table S1. A significant increase in the number of AMD prevalent cases as reported globally in different and age groups from 364.02 thousand in 1990 to 805.75 thousand in 2021, representing a percentage change of 121%, and the YLDs cases increased by 91% from 30.29 thousand in 1990 to 57.80 thousand in 2021. However, the ASPR and ASYR decreased steadily from 1990 to 2021, with EAPC of −0.26 (95% CI: −0.31 to −0.02) and −0.94 (95% CI: −1.01 to −0.88), respectively, but an anomalous rise was shown in 2020 (Figure 1). The findings of the joinpoint regression analysis on the prevalence and YLDS of AMD are illustrated in Figure 2. Joinpoint regression analysis identified four joinpoints for ASPR (1995, 1999, 2005, and 2013) and ASYR (1995, 1999, 2004, and 2018) during 1990–2021. Accordingly, ASPR and ASYR were divided into four segments, and each showing distinct changes in annual percent change (APC). Overall, both indicators demonstrated a significant downward trend in both genders [ASPR AAPC: −0.17(−0.30, −0.03); ASYR AAPC: −0.70(−0.83, −0.58)], however these trends varied across distinct join points. Notably, the ASPR of AMD showed an upward trend from 1990 to 1995, from 1999 to 2005, and from 2013 to 2021, and the ASYR of AMD exhibited an upward trend from1990 to 1995 (Figure 2).

**Table 1:** The Prevalence of AMD cases and rates in 1990 and 2021 across 27 regions, and the trends from 1990 to 2021.

Location	Prevalent cases			Prevalence rates		
	1990 (95% UI)	2021 (95% UI)	Percentage change (100%)	1990_per 100,000 (95% UI)	2021_per 100,000 (95% UI)	EAPC (95% CI)
Global	3,640,180 (3,037,098,4,353,902)	8,057,521 (6,705,284,9,823,238)	1.21 (1.14,1.29)	99.5 (83.16,118.04)	94 (78.32,114.42)	−0.26 (−0.31, −0.22)
Low SDI	286,203 (236,098,342,481)	623,529 (507,719,765,708)	1.18 (1.09,1.28)	144.7 (120.56,171.58)	139.92 (114.54,171.01)	−0.23(−0.28, −0.18)
Low-middle SDI	750,883 (613,961,907,402)	1,403,317 (1,142,219,1,725,627)	0.87 (0.79,0.95)	138.32 (114.13,165.93)	104.67 (85.46,127.94)	−1.03(−1.16, −0.91)
Middle SDI	1,041,645 (862,866,1,275,940)	2,790,576 (2,301,858,3,415,653)	1.68 (1.59,1.78)	114.58 (94.89,138.84)	107.56 (88.68,131.24)	−0.33(−0.39, −0.26)
High-middle SDI	917,446 (772,003,1,089,793)	2,103,144 (1,762,018,2,558,108)	1.29 (1.19,1.4)	100.42 (84.93,118.77)	104.55 (87.88,126.6)	0.12 (0.05, 0.18)
High SDI	641,152 (536,445,759,449)	1,132,042 (951,466,1,351,423)	0.77 (0.71,0.82)	56.98 (47.61,67.51)	48.43 (40.55,57.77)	−0.57(−0.64, −0.51)
Andean Latin America	22,193 (17,797,27,524)	65,055 (53,070,79,977)	1.93 (1.71,2.14)	118.47 (95.5,146.51)	113.6 (92.6,140.13)	−0.14(−0.26, −0.02)
Australasia	10,215 (8,383,11,992)	22,448 (18,261,26,698)	1.2 (1.06,1.34)	44.46 (36.72,52.45)	37.76 (30.65,44.98)	−0.48(−0.55, −0.42)
Caribbean	6,517 (5,220,8,233)	12,781 (10,161,15,899)	0.96 (0.88,1.05)	26.1 (20.88,32.83)	23.58 (18.71,29.36)	−0.28(−0.29, −0.26)
Central Asia	33,464 (26,591,42,068)	54,838 (43,829,69,066)	0.64 (0.57,0.71)	77.2 (61.54,96.52)	74.59 (59.75,93.95)	−0.15(−0.17, −0.13)
Central Europe	94,200 (76,937,114,526)	142,915 (116,166,177,362)	0.52 (0.46,0.58)	64.32 (52.9,77.73)	60.3 (49.02,74.97)	−0.27(−0.3, −0.24)
Central Latin America	42,532 (34,522,51,779)	122,677 (99,737,152,792)	1.88 (1.77,1.99)	57.92 (47.41,70.66)	50.91 (41.44,63.41)	−0.34(−0.36, −0.31)
Central Sub-Saharan Africa	5,248 (4,075,6,690)	13,289 (10,465,17,021)	1.53 (1.41,1.67)	31.73 (25.32,39.93)	33.45 (26.83,42.3)	0.23 (0.2, 0.26)
East Asia	894,592 (733,036,1,106,225)	2,680,112 (2,212,332,3,300,050)	2 (1.88,2.11)	118.72 (97.65,144.26)	121.81 (100.23,148.4)	−0.04(−0.18, 0.11)
Eastern Europe	73,045 (59,265,88,601)	89,364 (73,086,108,175)	0.22 (0.18,0.27)	27.07 (22.15,32.65)	24.51 (20.03,29.61)	−0.34(−0.39, −0.29)
Eastern Sub-Saharan Africa	82,571 (68,091,98,506)	166,626 (134,628,199,962)	1.02 (0.92,1.13)	129.97 (106.45,152.97)	116.04 (94.08,138.2)	−0.12(−0.21, −0.03)
High-income Asia Pacific	44,117 (36,507,53,266)	117,975 (98,416,142,412)	1.67 (1.46,1.91)	23.69 (19.74,28.57)	20.57 (17.16,24.97)	−0.43(−0.5, −0.36)
High-income North America	110,353 (92,081,130,296)	196,186 (163,871,234,253)	0.78 (0.73,0.82)	29.77 (24.9,35.14)	27.3 (22.77,32.6)	−0.34(−0.49, −0.19)
North Africa and Middle East	281,009 (228,122,337,967)	726,411 (588,700,890,108)	1.59 (1.46,1.71)	186.36 (151.99,221.21)	177.95 (144.8,218.13)	−0.17(−0.23, −0.11)
Oceania	904 (703,1,157)	2027 (1,580,2,586)	1.24 (1.13,1.36)	37.83 (30.17,46.91)	32.84 (26.1,41.15)	−0.36(−0.41, −0.31)
South Asia	798,545 (649,510,974,217)	1,583,589 (1,286,973,1,959,323)	0.98 (0.89,1.08)	157.88 (129.3,191.03)	113.88 (93.23,139.4)	−1.29(−1.48, −1.1)
Southeast Asia	212,794 (173,989,256,005)	465,204 (378,307,560,797)	1.19 (1.05,1.33)	96.28 (79.45,114.03)	77.01 (63.35,92.62)	−0.86(−0.92, −0.8)
Southern Latin America	16,574 (13,183,20,343)	29,421 (23,601,36,157)	0.78 (0.67,0.89)	38.04 (30.42,46.34)	32.32 (25.89,39.45)	−0.52(−0.55, −0.49)
Southern Sub-Saharan Africa	9,656 (7,765,11,927)	22,287 (18,071,27,802)	1.31 (1.24,1.38)	40.87 (33.37,50.23)	44.6 (36.73,54.69)	0.38 (0.31, 0.46)
Tropical Latin America	67,037 (54,762,82,525)	203,080 (166,748,248,976)	2.03 (1.91,2.15)	81.43 (67.39,98.71)	80.3 (65.95,98.34)	0.16 (0.02, 0.29)
Western Europe	652,170 (545,680,765,662)	939,465 (785,445,1,112,672)	0.44 (0.38,0.51)	107.4 (89.61,126.02)	85.55 (71.62,101.13)	−0.77(−0.8, −0.73)
Western Sub-Saharan Africa	182,444 (148,196,222,903)	401,771 (319,138,497,374)	1.2 (1.12,1.28)	226.01 (185.74,273.72)	229.48 (185.45,281.21)	−0.2(−0.3, −0.1)

AMD, age-related macular degeneration.

### 3.2 Region and country level

Overall, the number of prevalent and YLDs AMD cases in most regions and countries increased, while the prevalence and YLD rates exhibited the opposite trend (Table 1; Supplementary Tables S2–S4). Regionally, the highest ASPR and ASYR was observed in Western Sub- Saharan Africa (229.48 per 10,000, 95% UI: 185.45 to 281.21) and North Africa and Middle East (14.04 per 10,000, 95% UI: 9.6 to 9.61) in 2021, respectively. In contrast, the lowest was observed in High-income Asia Pacific (23.69 per 10,000, 95% UI:19.74 to 28.57) and Central Sub-Saharan Africa (2.02 per 10,000, 95% UI:1.36 to 2.89) in 2021 (Supplementary Figures S4A,B). Furthermore, South Asia and Southeast Asia were the top two regions with significantly decreasing trends of AMD over the past three decades with the EAPC of −1.29 (95% CI: 0–1.48 to −1.1) and −0.86 (95% CI: −0.92 to −0.8) for ASPR and 1.81 (95% UI: −1.99 to −1.63) and −1.38 (95% UI: −1.45 to −1.31) for ASYR. Moreover, the ASPR and ASYR of AMD increased contrast in some countries in Central Sub-Saharan Africa and Southern Sub-Saharan Africa, and the EAPC of ASPR were 0.23 (95% CI: 0.2 to 0.26) and 0.38 (95% CI: 0.31 to 0.46), and of ASYR were 0.12 (95% UI: 0.05 to 0.19) and 0.27 (95% UI: 0.13 to 0.42; Supplementary Figures S4C,D).

At national levels, the highest ASPR and ASYR were seen in Iran (Islamic Republic of; 346.81 per 100,000, 95% UI: 291.39 to 411.32) and Kenya (34.32 per 100,000, 95% UI:22.14 to 49.32) in 1990, while in 2021 the highest ASPR and ASYR worldwide was noted in Nepal (399.22 per 100,000, 95% UI: 315.31 to 507.37) and Iran (Islamic Republic of; 25.02 per 100,000, 95% UI:17.16 to 34.88). Besides, the prevalence and YLDs rate in most countries exhibited a downward trend from 1990 to 2021. Malaysia, Thailand, India and Iceland were the four leading countries with significantly decreasing trends over the past three decades, and the upward trends can be observed in some countries such as Niger and Benin. However, the cases of prevalent and YLDs still showed opposite trend that only 1% of countries exhibited a decrease in the cases of prevalent and YLDs such as Tokelau (−8, 95% CI: −15% to 0) and Niue (−35, 95% CI: −44 to −24%; Supplementary Tables S3, S4).

### 3.3 Age pattern

**Figure 3:**
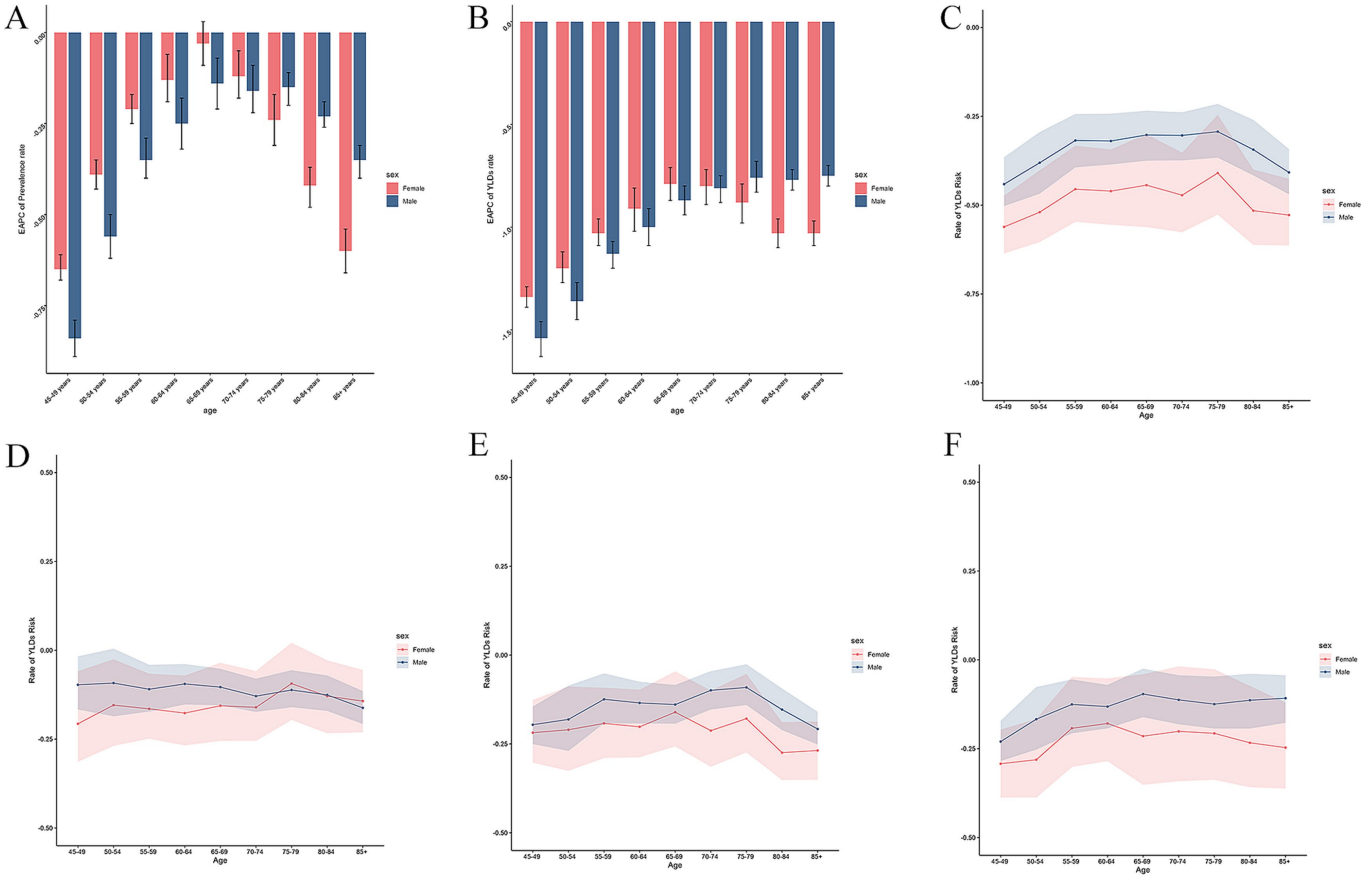
Temporal trend of AMD burdens from 1990 to 2021 by sex. **(A)** EAPC of prevalence rate 1990 to 2021. **(B)** EAPC of YLDs rate 1990 to 2021. **(C)** Rate of AMD burden caused by risk factors in different age groups from1990-2021. **(D)** Rate of AMD burden caused by risk factors in different age groups from1990-2000. **(E)** Rate of AMD burden caused by risk factors in different age groups from2000-2010. **(F)** Rate of AMD burden caused by risk factors in different age groups from2010-2021. AMD, age-related macular degeneration; YLDs, years lived with disability; EAPC, estimated annual percentage change.

Globally in 2021 the number of prevalent and YLD cases due to AMD exhibited a gradual increase with age, reaching a peak at the age of 65–69, and subsequently a decline in cases was observed (Supplementary Figures S1A,B). A similar trend that was also noted in 1990 (Supplementary Figures S1C,D). In 2021, an upward trend can be seen with age increasing in the crude rate of prevalence and YLDs (Supplementary Figures S1A,B), and the highest prevalence and YLDs rate was observed in the over 85 years age group (1349.99 per 100,000, 95% UI: 1102.32 to 1652.2 and 115.44 per 100,000, 95% UI: 77.49 to 160.95; Supplementary Tables S4, S5). And it can also be observed that the rate of the increase accelerates obviously after the age of 85 years old in 2021 (Supplementary Figures S1A,B), however it was less obvious than 1990 (Supplementary Figures S1C,D). The crude rate in each age group decreased from 1990 to 2021, and the 45–49 age group demonstrated the rapidest decline in prevalence and YLD rate (EAPC: -1.43, 95% CI: −1.5 to −1.36). Apart from that, we also observed that the decline speed of prevalence and YLD rate from 1990–2021 exhibited a gradual deceleration with increasing age, with a lower absolute EAPC in the 60–64, 65–69, and 70–74 age groups, and then gradually decreased at a faster rate with increasing age, reaching a higher level of absolute EAPC in the over 85 age group (Figures 3A,B). However, from 1900 to 2021, the number of AMD prevalence and YLDs cases exhibited an upward trend in each age group (Figures 1B,C).

### 3.4 Sex and age pattern

Overall, females exhibited a higher rate of prevalence and YLDs compared to males in each age group in the past 3 decades (Supplementary Figures S1A,B). The disparity between the sexes became more pronounced with age, however the disparity narrowed compared to 1990 (Supplementary Figures S1C,D). It can be observed the prevalence and YLDs rate increasing with age in both males and females. Furthermore, we can also observed females increased faster than males in the age group above 80 years old. Apart from that, as mentioned above, the rate of prevalence and YLDs declined from 1990 to 2021 in both sexes. And we found that the decline of the crude rate of prevalent and YLDs cases is faster in females than in males in the age groups over 65 years old, conversely, in other age groups it was slower in males. Besides, as age increases, the rate of decline in both sexes exhibit a process of slowing down and then accelerating, with a faster rate of decline observed in the older age groups versus the middle-aged groups (Figures 3A,B).

### 3.5 SDI and age pattern

**Figure 4:**
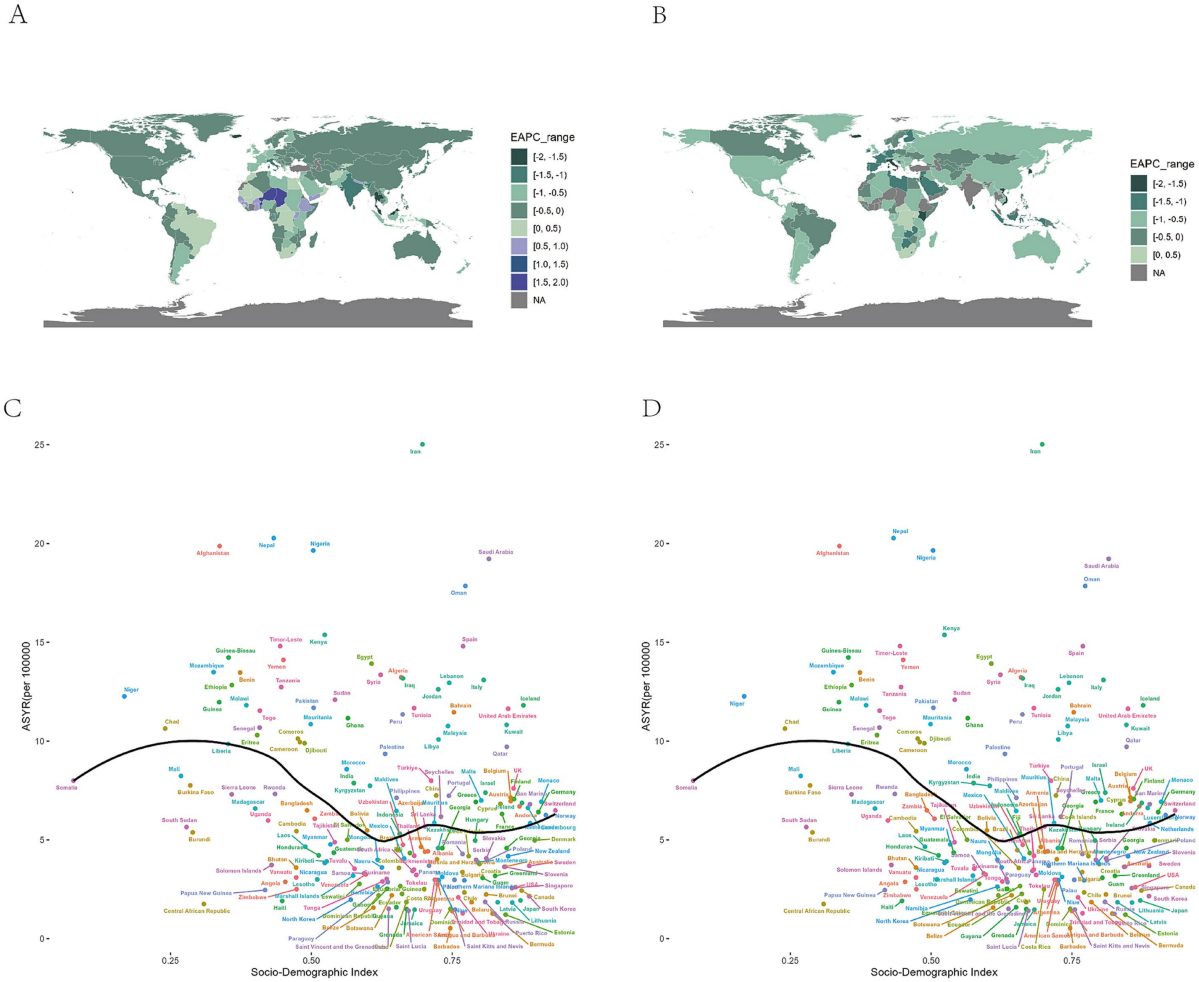
Global burden of the prevalence and YLDs of AMD by SDI and countries. **(A)** The EAPC of ASPR for AMD from 1990 to 2021. **(B)** The EAPC of ASYR for AMD from 1990 to 2021. **(C)** The black line represents the average expected relationship between SDIs and age standardized prevalence rates for AMD based on values from 204 countries and territories in 2021. **(D)** The black line represents the average expected relationship between SDIs and age-standardized YLD rates for AMD based on values from 204 countries and territories in 2021. AMD, age-related macular degeneration; SDI, socio-demographic index; ASPR, age-standardized prevalence rate; ASYR, age-standardized YLD rate; YLDs, years lived with disability; EAPC, estimated annual percentage change.

The highest number of prevalent and YLDs of AMD were observed in the middle SDI region (279.06 thousand, 95% UI: 230.19 to 341.57and 18.79 thousand, 95% UI: 12.92 to 25.91), accounting for one-third of the global total in 2021 (Supplementary Figures S2C,D). Furthermore, the highest ASPR and ASYR were shown in the low SDI regions (139.92 per 100,000, 95% UI: 114.54 to 171.01) and (10.08 per 100,000, 95% UI: 6.91 to 13.86; Supplementary Figures S2A,B). From 1990 to 2021, it was observed that the middle SDI region showed the largest percentage change. In contrast, the high SDI region exhibited the smallest percentage change (Supplementary Figure S2F). Notably, the ASPR and ASYR showed a decreasing trend in in most SDI regions, except for the ASPR in high-middle SDI region (Supplementary Figure S2E). The low-middle SDI region was the top region decreasing in both ASPR and ASYR (EAPC: −1.03, 95% CI: −1.16 to −0.91 and −1.48,95% CI: −1.6 to −1.36), and the ASPR and ASYR of low SDI region decreasing slower than other SDI regions (Table 1; Supplementary Figure S2E). The relationship between ASPR and ASYR and SDI in 204 countries and territories in 2021 shown in Figure 4. In countries, the burden of AMD in Pakistan, Iran, Afghanistan, and Yemen and so on exceeds expectations, while in some places such as South Korea, Russia, US, and Ethiopia, the burden is lower than anticipated. ASPRs and ASYRs in developing countries and regions were significantly higher than those in developed countries and regions. Regions such as Tropical Latin America and Western Europe have a higher burden than expected, while regions such as Eastern Sub-Saharan Africa, Andean Latin America, and high-income Asia Pacific have a lower burden than expected (Table 1; Supplementary Table S1).

**Figure 5:**
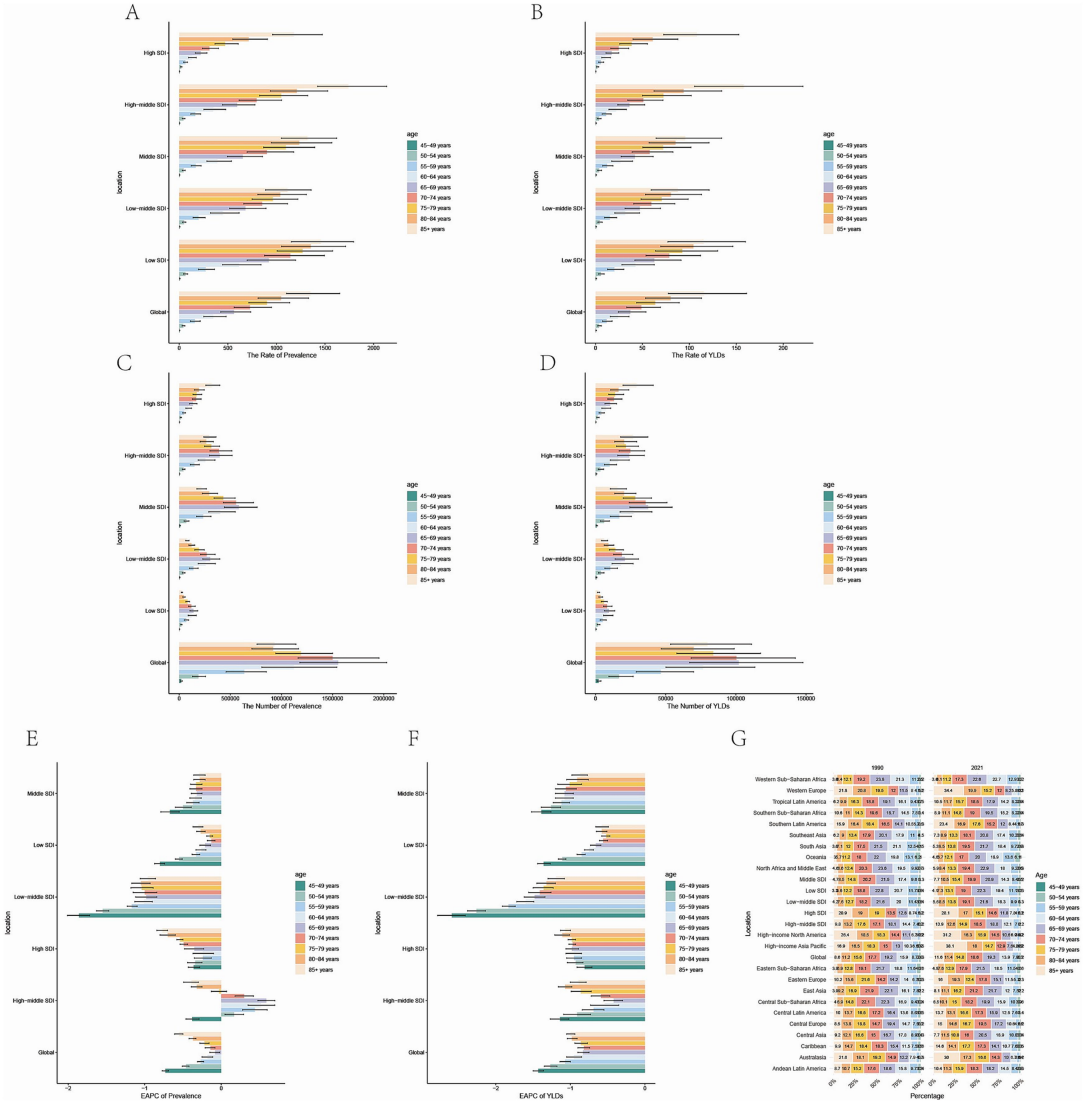
Temporal trend of AMD burdens in different age groups in SDI regions. **(A)** Prevalence rate per 100,000 population in 2021. **(B)** YLDs rate per 100,000 population in 2021. **(C)** Number of prevalent cases in 2021. **(D)** Number of YLDs cases in 2021 **(E)** EAPC of prevalence rate 1990 to 2021. **(F)** EAPC of YLDs rate 1990 to 2021. **(G)** Percentage of prevalent cases by age group, EAPC, Estimated Annual Percentage Change. AMD, age-related macular degeneration; YLDs, years lived with disability; SDI, socio-demographic index; EAPC, estimated annual percentage change.

Additionally, the highest number of prevalence and YLDs cases and the highest crude prevalence and YLDs rate were observed in the 65–69 age group of middle SDI region and the over 85 age group of the high middle SDI region (Figures 5A–D). Most SDI regions showed a downward trend of ASPR and ASYR from 1990 to 2021, and the 45–49 age group in the low-middle SDI region had the rapidest decrease. However, some age groups in the high-middle SDI region exhibited an upward trend of ASPR (Figures 5E,F). Besides, the percentage of the prevalence and YLDs rate in different age groups were shown in Figure 5G and Supplementary Figure S3E. It can also be observed that there was no obvious alteration in the proportion of individuals across various age demographics within the low SDI region. Conversely, the high SDI region exhibited a considerable rise in the proportion of individuals within the 70–74 age group and the over 85 age group in2021 compared with 1990, and we observed the percentage of AMD in low SDI region is higher in the 55–74 age group than the older age groups, and in high SDI region, the older age groups’ percentage are higher.

### 3.6 Risk factor

The management of risk factors can be reflected in part by changes in the rate of YLDs, with a decrease in the rate reflecting better control of risk factors. We observed diverse trends for the decrease between male and female in different age group and different periods. Compared to the 1990–2000 and 2000-2010, the YLDs rate of male in over 85 age group is higher from 2010 to 2021 (Figures 3C–F).

## 4 Discussion

This study provided the data on the prevalence and YLDs of AMD at global, regional, and national levels from 1990 to 2021, and further presented a comprehensive and detailed description based on trend analyses, then revealed disparities of disease burden by age, sex and socioeconomic level. Apart from that, the role of age factors across SDI regions and across gender can also be observed in our study, which is mentioned in other study related to AMD rarely. In our study, significant differences in the disease burden for AMD were observed across regions and countries. Although the findings of this study indicated a downward trend in the ASPR and ASYR of AMD from 1990 to 2021, the number of AMD prevalent and YLDs cases increased, which may be caused by the global population growth and population ageing. The global population aged 65 and over in 2000 was 18 percent, while by 2050 it will reach 38 percent. AMD disease ends with loss of vision (16). The phenomenon of vision loss is a significant public health and socio-economic concern, impacting not only economic prospects but also educational opportunities. To eradicate all preventable vision loss and enhance global eye health services, particularly in addressing age-related eye diseases such as macular degeneration and cataracts, as well as treatable blindness, the International Council of Ophthalmology (ICO) and the World Health Organization (WHO) initiated the “Vision 2020: The Right to Sight” initiative in 1999 (17). As the leading cause of visual disability among patients over 55 years (18), understanding the temporal and geographic trends in the prevalence and burden of AMD, as well as exploring its related risk factors, will help individuals, decision-makers, and health managers take better action.

Our findings are consistent with those of a previous report that analyzed the GBD data between 1990 and 2019, which also found the highest number of prevalent and YLD cases in East Asia, followed by South Asia (9). These regions have also demonstrated an upward trend over the past three decades, primarily attributable to the substantial population of China and India (19). In contrast to the GBD study in 2019, the ASPR and ASYR in East Asia exhibited a downward trend between 1990 and 2021 (9). This phenomenon may be associated with the emergence of more comprehensive public health policies in certain regions, such as China. In accordance with the “Healthy China 2030” initiative and with the objective of further promoting the high-quality development of eye health during the 14th Five-Year Plan period and enhancing people’s eye health, the National Health Commission has formulated the 14th Five-Year National Eye Health Plan (2021–2025) (20). The highest ASPR and ASYR observed in Western Sub-Saharan Africa, North Africa, and Middle East may be attributed to their locations with higher UV intensity and diets with fewer vegetable grains (21). Individualized public health policies tailored to the geo-cultural and dietary characteristics of the different regions are particularly important for reducing the global burden of AMD. Notably, concerns should be raised not only in regions with the highest burden currently, but also in regions with the highest increasing magnitude over the last decades. We observed that Southern Sub-Saharan Africa and Central Sub-Saharan Africa exhibited an upward trend in the past 30 years contrary to most regions, as previously reported. This phenomenon can be attributed to the region’s high population of tribal communities, which is compounded by the generally lower economic development, health status and worse risk factors control (22, 23). It also reveals the need for relevant health policy development in the region. Although AMD is a global disease, its epidemiological and economic impacts differ substantially between developed and developing countries. Population-based studies in developing regions, such as India and Iran, indicate substantial prevalence and late-stage disease among older adults, with prevalence increasing sharply with age, from approximately 8% in the 60–69 years group to nearly 19% in the 80–84 years group in Iran (24). By contrast, in high-income settings such as the United States, Europe, and Singapore, AMD is generally detected earlier, and age-standardized prevalence has declined in recent decades, particularly in older age groups (8, 25). Treatment patterns and access also differ: anti-VEGF therapy is widely available and more consistently applied in high-income countries, whereas in low- and middle-income countries, limited accessibility and financial constraints restrict treatment coverage, particularly among older patients (26). These findings highlight the widening inequalities between developed and developing countries, where the former primarily face challenges of managing long-term treatment in aging populations, while the latter continue to struggle with delayed diagnosis, limited accessibility, and constrained treatment availability.

Although prior studies have identified numerous risk factors associated with AMD progression, age is a particularly salient factor (27). Consistent with prior research, we observed that the prevalence of AMD increases with age, a phenomenon attributed to the accumulation of oxidative damage, decline in cellular repair capacity, activation of chronic inflammation, vascular aging, and other mechanisms that collectively result in damage to the retinal epithelium with age, ultimately leading to structural damage and loss of function in the macular (28–30). And the phenomenon that the more obvious increasing trend in the age above 80 years indicates the necessity to pay more attention to this age group. As for the reason why the 65–69 age group has the most patients may be caused by the base population. And this finding reflects the effectiveness of AMD education and prevention in older patients. Furthermore, the study revealed that the prevalence and YLDs rate of AMD patients in the EAPC exhibited a decline over the past three decades, with a more pronounced decline observed in the 45–49 age group and the over 85 age group, while in the intermediate age group was more gradual. To find the reason, we analyze the risk factor-related indicators in the GBD subsequently and it revealed similar trends in risk factors ‘burden across age groups over the past three decades (Figure 3C). GBD2021 provides the burden of disease associated with only one risk factor, smoking. Smoking is the characterized risk factor for AMD, with the risk multiplied by 2.5 to 4.5 in current smokers, and decreasing with time from smoking cessation (31, 32).

In addition to environmental and age factors, the economic development of a region also influences the burden of AMD. Understanding the distribution of AMD across SDI regions is crucial for effective healthcare planning and resource allocation. Generally, higher levels of SDI are associated with more robust healthcare systems and higher quality of medical services, which in turn leads to a reduction in disease burden. This phenomenon partially explains the higher ASPR and ASYR observed in low SDI regions compared to other SDI levels within our study population. However, consistence with former studies. The middle SDI region exhibited the highest AMD prevalent and YLDs cases (10), and it mainly caused by the substantial population of China and India (19). Furthermore, the prevalence of high middle SDI region, although not currently the highest prevalent, is on the rise, indicating a higher AMD burden in this region. The most plausible hypothesis for this result is that with the economic development of the high middle SDI region, the reported increase in AMD-related risk factors and disease diagnoses, rapid urbanization and industrialization have accelerated changes in people’s lifestyles, including lack of physical activity, unhealthy dietary habits, and an increase in systemic disease illnesses, such as hypertension and diabetes mellitus (33). These changes, in turn, undermine the control of disease through sustained growth of the economic growth and social development to control the burden of disease. This phenomenon should also be concerned.

In addition, our study focused on the variability of AMD disease burden across different age groups in each SDI region. It is obvious that the crude prevalence and YLDs rate increase by age no matter in any SDI region and decreased from 1990 to 2021 no matter in males and females. However, we observed a significantly faster decline in ASPR and ASYR among younger AMD patients compared to older patients in regions with lower SDI levels from 1990–2021. Conversely, in regions with higher SDI levels, the speed of decline in disease burden among older patients was significantly faster than that among younger patients. This finding is inconsistent with the results of previous studies. We hypothesize that age is the most significant risk factor for AMD, and it generally results in a lower disease burden in younger patients than in older patients in the absence of non-health policy interventions. This is somewhat consistent with the trend of disease burden in regions with a low SDI level. However, in regions exhibiting higher levels of development, the emphasis of AMD and eye health-related health policies on the senior population, coupled with the increased exposure to social pressure and risk factors for middle aged even younger age group, has resulted in a trend of a faster decline in AMD disease burden among older individuals compared to younger ones (34). For example, The National Eye Institute (NEI) in the United States proposed Strategic Plan: Vision for the Future 2021–2025 identifying AMD as a priority research area to support the use of artificial intelligence (AI) in fundus screening (34). This phenomenon has underscored the necessity for the relevant authorities to direct their attention toward the formulation of health policies pertaining to AMD in relatively younger age groups. Besides, we also found differences in the age composition of patients in different SDI regions, with higher SDI regions having a larger proportion of older patients and lower SDI regions having a larger proportion of people in the 55–74 age group, which may be related to the fact that the higher the level of SDI, the more serious the phenomenon of aging. It implies a higher need for public health policies.

In accordance with prior research, our study documented a higher prevalence and number of female patients than male patients in 1990 and 2021 (9, 10). This gender disparity in the disease may be attributable to the prolonged life expectancy of women, as well as to the heightened chronic inflammatory response in women during the aging process and the elevated pigment density of the macular area in women, and the discrepancy in light absorption affects photodamage sensitization (33–36). Besides it has been posited that the expression or mutation of certain genes associated with AMD, such as complement factor CFH and ARMS2/HTRA1, may be influenced by sex hormones (32). These mechanisms could potentially contribute to a heightened susceptibility to the pathologic phenotype in women. Furthermore, our study revealed a downward trend in the prevalence of AMD among both sexes over the past three decades, concomitant with an upward trend in the number of cases. We also found that the crude rate of prevalence and YLDs increased with age for each sex, but is consistently higher for females than for males, and the difference is more pronounced with age. Besides, the rate though decreased in all genders from 1990 to 2021, the downward trend varied across genders. Specifically, descent rate of male patients aged below 65–69 years exceeded that of female patients, while female patients aged 70 years and above exhibited a descent rate that surpassed that of male patients. The examination of the shift in the aggregate trend of AMD patients’ number across both sexes may be attributable to the expansion of the population base (16), and the decline in ASR from 1990–2021 showed the initial efficacy of the management of associated diseases. To further analyze the reasons for the observed differences across various age groups, a correlation analysis of risk factors for age-related macular degeneration was conducted. The results indicated that the level of control of smoking risk factors was significantly higher in women compared to men, especially in the relatively advanced age group. Women are more sensitive than men to the risk factor of smoking, and they have a higher rate of decline in disease burden than men at the same level of risk factor control. This discrepancy may be attributed to the higher levels of oxidative stress observed in female smokers under the influence of estrogens declining after menopausal (37, 38), resulting in more intense retinal damage.

Furthermore, over the past three decades, we found that the AMD burden attributable to smoking showed distinct age-specific patterns between men and women, which has rarely been highlighted in previous epidemiological studies. In recent years, changes in societal attitudes and behaviors have increased the percentage of smokers in the population, especially in the female population, and this, combined with a higher susceptibility to AMD in female smokers, has led to an increase in the burden of disease due to smoking in female AMD patients in recent years compared to the 1990’s. Furthermore, we have observed that smoking control has been poorer in male patients over 85 years of age in the last 10 years compared with the previous 20 years, indicating a need for continued attention to risk factor management in these populations. Epidemiological studies quantifying the disease burden of AMD due to risk factors are limited. The findings of this study indicate that effective management of the risk factor associated with smoking plays a significant role in mitigating the AMD disease burden and it can offer supplementary recommendations for the development of public health policies and guidelines concerning AMD to some extent.

## 5 Limitation

The GBD studies provide detailed estimates of the global burden and risk factors for AMD-related deaths but have certain limitations. These common shortcomings are explained in detail in many previously published GBD studies. In short, data collected from various regions and countries may vary considerably in terms of quality, comparability, accuracy, and degree of missingness, which inevitably leads to some bias in the estimates, even when the data are adjusted as much as possible using many statistical methods. Besides, this study combined point estimates from Bayesian modeling provided by the Global Burden of Disease with frequentist methods such as Joinpoint regression in trend analysis. Although this approach is widely adopted in GBD-related research, it does not directly incorporate the inherent posterior uncertainty of Bayesian models, necessitating caution in interpreting results. Future studies may consider employing Bayesian hierarchical models or empirical Bayesian trend methods to more comprehensively integrate uncertainty. Moreover, we were unable to distinguish the contributions of different AMD subtypes, including atrophic AMD and exudative AMD, to the overall mortality burden. In addition, the lack of data on relevant risk factors other than smoking, such as inadequate intake of omega3 from a high-fat diet, obesity, and ultraviolet exposure, limited the analysis of overall risk factors, future research should integrate original cohort data or other epidemiological databases to examine additional risk determinants beyond smoking. Finally, our analysis of the burden of AMD was conducted at the regional and national level, without further analysis of local characteristics, such as urban–rural differences.

## 6 Conclusion

In conclusion, although the prevalence of AMD and YLD rates are declining, the burden of disease caused by AMD cannot be ignored due to the increase in the population base and the aging of the population; the burden of disease is still greater in people over 80 years of age and in less developed regions, but it is worth noting that the proportion of patients aged 65–80 years in the AMD population is significantly higher, especially in countries with lower levels of SDI. AMD is showing a trend of “relative youthfulness.” In addition, although the burden of smoking as a risk factor was generally lower in women than in men over the past 30 years, the burden of disease was higher in women, and this gender gap increased with age. Together, these results highlight the significant challenges in the control and management of AMD, including the increase in the number of cases and the unequal distribution of disease across age groups and sexes globally, which may guide better public health policy and allocation of healthcare resources. The priority population to be managed for AMD remains the older population, but the age range should be broadened, and health policies should be developed for all people over 65 years of age. In addition to this, regions should consider the local gender composition and the level of SDI development to propose targeted strategies for different age groups. This also suggests that it would be useful to regularly update disease status within the framework of GBD and provide timely assessments.

## Data Availability

The original contributions presented in the study are included in the article/Supplementary material, further inquiries can be directed to the corresponding author.

## Footnotes

^1^ https://ghdx.healthdata.org/gbd-2021/sources
