# Supplementary material for: Global burden of age-related macular degeneration (1990–2021): trends, age-sex disparities, and socioeconomic dynamics from the GBD study
Source: Front Public Health. 2025 Oct 23;13:1594672. doi: 10.3389/fpubh.2025.1594672 (PMC12588936; doi:10.3389/fpubh.2025.1594672)
Supplement: Supplementary file 2 [file Data_Sheet_1.pdf]

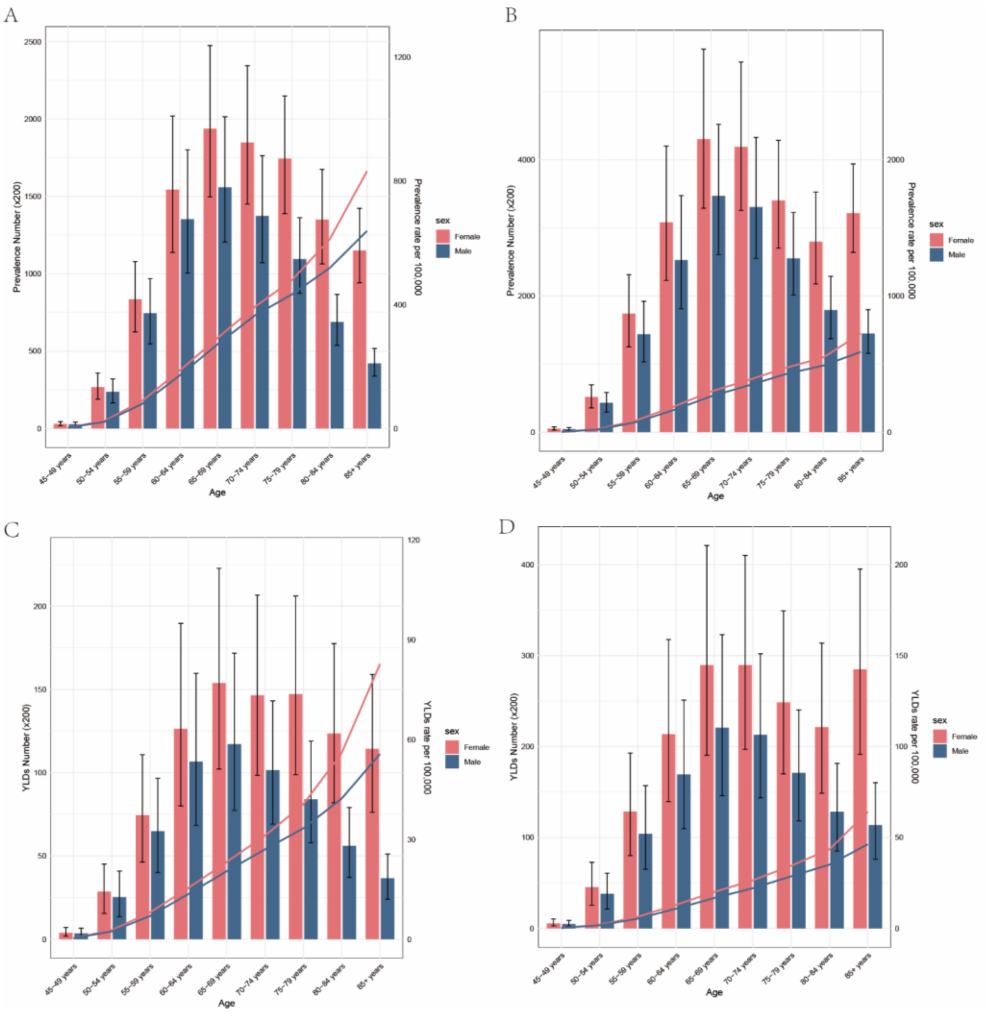

Figure.S1. Global burden of the prevalence and YLDs of AMD by age and sex in 1990 and 2021. (A) The number of cases and crude prevalence rates of AMD in 2021. (B) The number of cases and crude YLDs rates of AMD in 2021. (C) The number of cases and crude prevalence rates of AMD in 1990. (D) The number of cases and crude YLDs rates of AMD in 1900. Red and blue dashed line indicates the upper and lower limits of the 95% uncertainty intervals (95% UIs) for females and males, respectively.

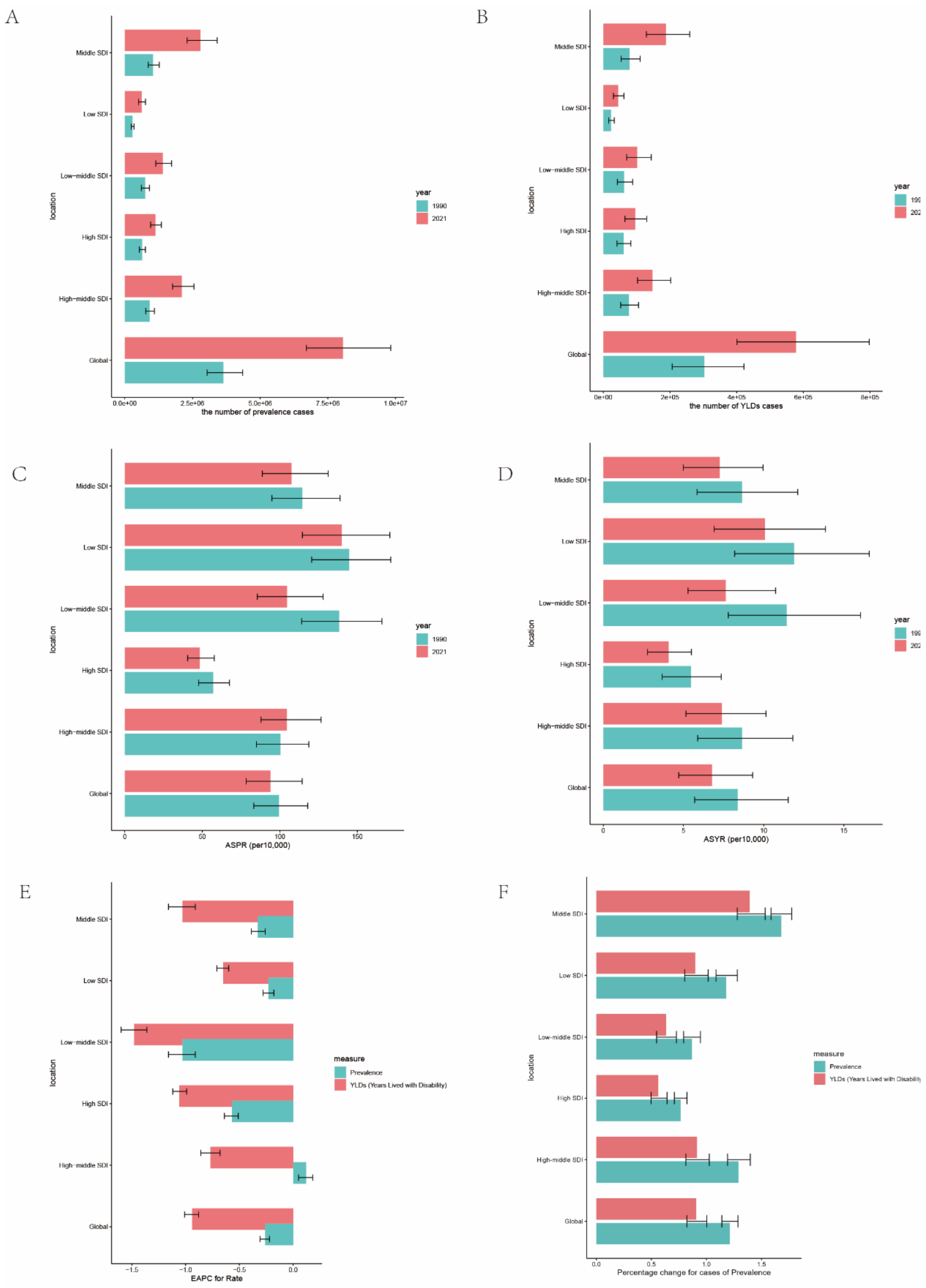

FigureS2. Temporal trend of AMD burdens in SDI regions in 1990 and 2021 (A) Prevalence rate per 100,000 population. (B)YLDs rate per 100,000 population (C) Number of prevalent cases. (D) Number of YLDs cases (E) EAPC of prevalence and YLDs rate 1990 to 2021. (F) Percentage changes of Prevalence and YLDs cases.EAPC, Estimated Annual Percentage Change.

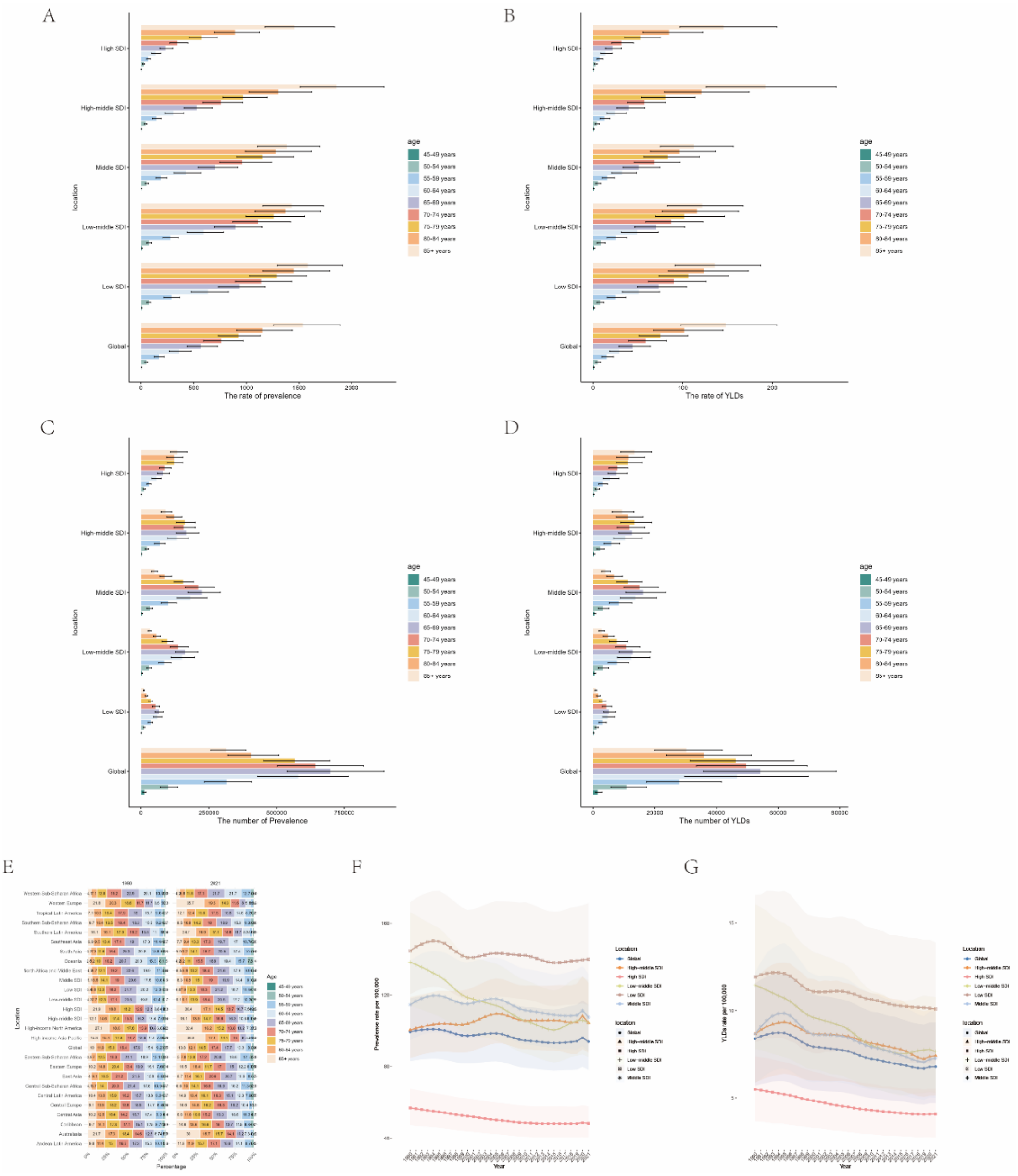

FigureS3. Temporal trend of AMD burdens in different age groups in SDI regions. (A) Prevalence rate per 100,000 population in 1990. (B) YLDs rate per 100,000 population in 1990. (C) Number of prevalent cases in 1990. (D) Number of YLDs cases in 1990. (E) Percentage of YLDs cases by age group. (F) The rates of prevalence from 1990 to 2021 in SDI regions. (G) The rates of YLDs from 1990 to 2021 in SDI regions.

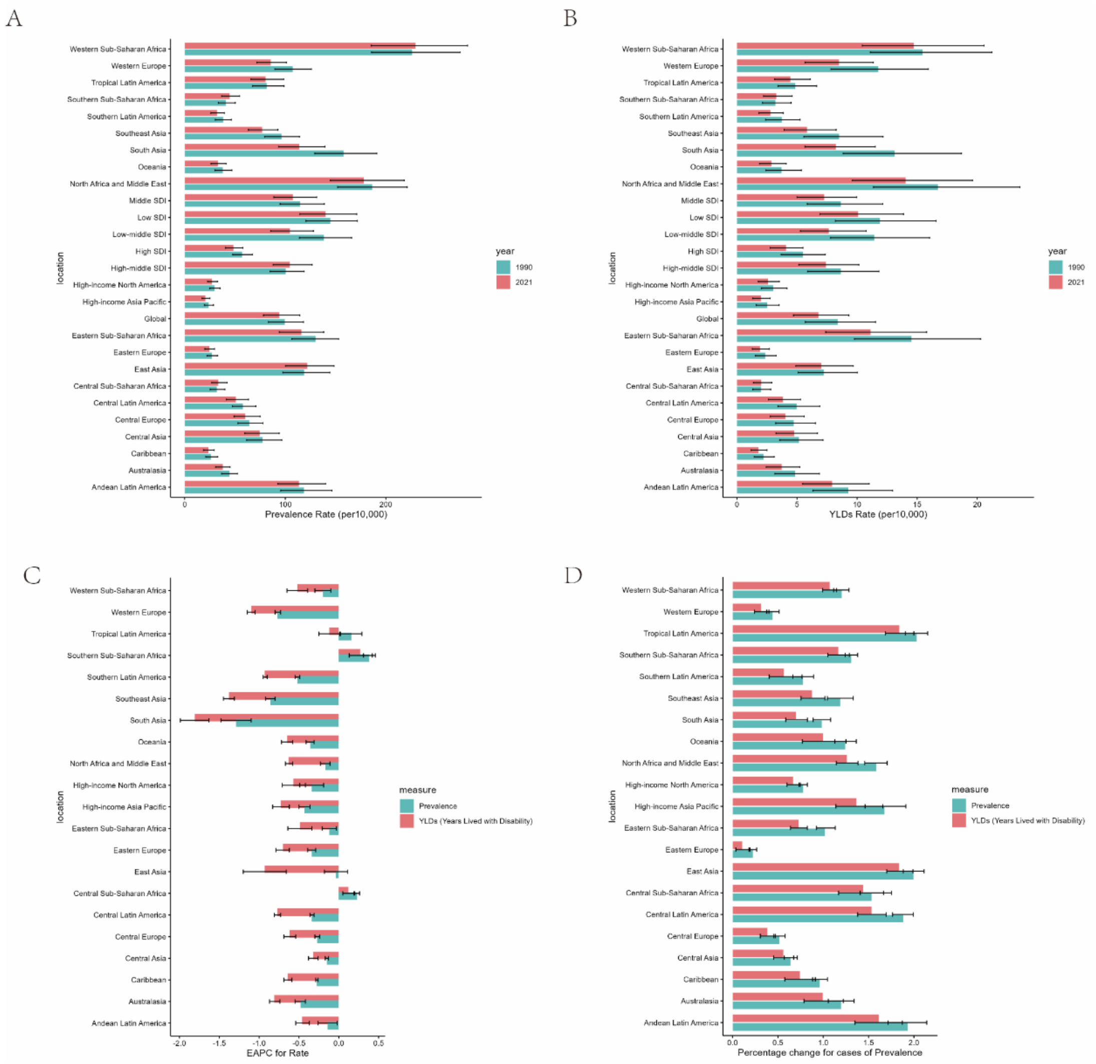

FigureS4. Temporal trend of AMD burdens in 27 regions in 1990 and 2021 (A) Prevalence rate per 100,000 population. (B)YLDs rate per 100,000 population. (C) EAPC of prevalence and YLDs rate 1990 to 2021. (D) Percentage change of Prevalence and YLDs cases.
